# CLEC4s as Potential Therapeutic Targets in Hepatocellular Carcinoma Microenvironment

**DOI:** 10.3389/fcell.2021.681372

**Published:** 2021-08-02

**Authors:** Yinjiang Zhang, Hongyun Wei, Lu Fan, Mingyan Fang, Xu He, Binan Lu, Zongran Pang

**Affiliations:** ^1^School of Pharmacy, Minzu University of China, Beijing, China; ^2^Key Laboratory of Ethnomedicine, Minzu University of China, Ministry of Education, Beijing, China; ^3^Morning Star Academic Cooperation, Shanghai, China; ^4^Department of Gastroenterology, Affiliated Hospital of Qingdao University, Qingdao, China

**Keywords:** hepatocellular carcinoma, tumor microenvironment, CLEC4s, immune, therapeutic targets

## Abstract

Immunosuppressive tumor microenvironment in hepatocellular carcinoma (HCC) is critical in tumor development. C-type (Ca^2+^ -dependent) lectin (CLEC) receptors, essential in innate pattern recognition, have potential regulatory effects on immune cell trafficking and modulatory effects on cancer cell activity. However, information on the expression and prognostic value of CLECs in HCC is scanty. Herein, we explored the potential role of CLECs in HCC based on TCGA, ONCOMINE, GEPIA, UALCAN, cBioPortal, Metascape, TRRUST, and TIMER databases. Results demonstrated a significantly higher mRNA level of CLEC4A and CLEC4L in HCC tissues than normal liver tissues. Contrarily, we found significantly low CLEC4G/H1/H2/M expression in HCC tissues. The IHC analysis revealed the following: Absence of CLEC4A/J/K/M in normal and liver cancer tissues; high CLEC4C expression in HCC tissues; low expression and zero detection of CLEC4D/E/H1/H2/L in HCC tissues and normal tissues, respectively. And the HepG2 and LX-2 were used to verify the expression level of CLEC4s via qRT-PCR *in vitro*. Furthermore, the expression of CLEC4H1 (ASGR1) and CLEC4H2 (ASGR2) exhibited a significant relation to clinical stages. However, the expression of CLEC4A, CLEC4D, CLEC4E, CLEC4J (FCER2), CLEC4K (CD207), CLEC4G, CLEC4H1, CLEC4M, and CLEC4H2 decreased with tumor progression. Patients expressing higher CLEC4H1/H2 levels had longer overall survival than patients exhibiting lower expression. Moreover, CLEC4A/D/E/J/K/G/H1/M/H2 had significant down-regulated levels of promoter methylation. The expression level of CLEC4s was correlated with the infiltration of B cells, CD8 + T cells, CD4 + T cells, macrophage cells, neutrophil cells, and dendritic cells. Functional analysis revealed the potential role of CLECL4s in virus infection, including COVID-19. Also, hsa-miR-4278 and hsa-miR-324-5p, two potential miRNA targets of CLEC4s, were uncovered. This article demonstrates that CLEC4 is crucial for the development of HCC and is associated with infiltration of various immune cells, providing evidence for new immunotherapy targets in HCC.

## Introduction

Hepatocellular carcinoma (HCC) is among the most causes of cancer-related deaths worldwide. Although surgical resection or ablation is regarded as the first-line treatment for early HCC, tumor recurrence within 5 years is estimated at 70%. Over the centuries, treatments for HCC include chemotherapy, target therapy, and immune checkpoint inhibitors (Falzone et al., 2018; Christofi et al., 2019). Although these treatments have significantly improved the survival rate of HCC patients, for advanced HCC, the current medical treatment is still not cost-effective (Zhuo et al., 2019). Treatment strategies for advanced HCC are still lacking, and Sorafenib as the first-line treatment option for advanced HCC has limited survival benefit in advanced HCC (Bhayani et al., 2015; Neureiter et al., 2019). Owing to the unclear mechanisms associated with the progression of HCC, this malignant tumor is intractable to therapeutic interventions, which warrants further studies to uncover new therapeutic targets.

Recently, the tumor microenvironment (TME) was found to play a key role in the development of HCC (Mroweh et al., 2020). TME constitutes immune cells and some products produced by cancer cells or immune cells. In a recent study, single-cell cytometry and transcriptome sequencing of TME in 13 HCC patients demonstrated that 17,432,600 immune cells, including tumor-associated CD4/CD8 double-positive T cells, and high IFN-γ/TNF-α levels were located at TME of HCC (Zheng et al., 2020). These immune cells could secret cytokines, such as interleukin-10, that potentially induced angiogenesis (Shiraki et al., 2011). Additionally, tumor-associated chemokines could drive tumorigenesis through polarization of immune subsets into pro-tumor phenotype or by recruiting T regulatory cells (Mantovani and Locati, 2013). Clinicians are emphasizing immunotherapy as a second-line approach to managing HCC (Abd El Aziz et al., 2020; Federico et al., 2020). Scholars have also tested several immune checkpoint blockers (ICBs) such as programmed cell death protein 1 (PD-1) and anti-cytotoxic T lymphocyte-associated antigen 4 (CTLA-4) in clinical trials (Lee et al., 2020; Pinter et al., 2020). Despite the approval of these agents for HCC treatment, the response rate ranges between15 and 23%, which is still low (Hato et al., 2014). This demonstrates how immunotherapy is faced with enormous hurdles.

C-type (Ca^2+^ -dependent) lectin (CLEC) receptors, involved in innate pattern recognition, are indispensable for several aspects of the immune system (Takeuchi and Akira, 2010). The CLEC family comprises numerous molecules, including CLEC1, CLEC2, CLEC4, etc. Compelling evidence shows that CLECs exert critical roles in activating and reshaping the immune system (Hoober et al., 2020). Particularly, CLEC4α3 was found to increase the infiltration of T cells into the spinal cord following nerve root injury (Lindblom et al., 2013). CLEC2 is mainly expressed in platelets or megakaryocytes, and it can adhere to cancer cells to induce the release of proinflammatory cytokines (Suzuki-Inoue, 2019). Consequently, the proliferation of gastric cancer cell lines was suppressed (Xu L. et al., 2020). Besides, CLEC2 promoted lung metastasis of osteosarcoma carcinoma through cell adhesion through interaction with podoplanin (Schlesinger, 2018; Ichikawa et al., 2020). In summary, CLECs are crucial in activating the immune response, despite that the mechanisms underlying the association of CLECs with the development of HCC through reshaping immune system is unclear. In this article, we aimed to explore the role of CLECs in HCC and evaluate their potential association with reshaping immune system in the development of HCC. A flow chart as shown in Supplementary Figure 9 summarized the overall steps for the target gene identification and analysis. First, we analyzed the mRNA levels of top under-expressed genes in HCC, and then we confirmed the expression of CLEC4s in HepG2 based on experiments and the CCLE database. Last, we analyzed the potential function in reshaping immune system, their interacted proteins and evaluation effects based on the public database.

## Materials and Methods

### Expression Profiling

To perform a genome-wide analysis of the expression of CLECs in HCC, data were extracted from UALCAN^1^ based on the TCGA database. In total, 371 HCC patients and 50 normal controls were enrolled for further analysis. Verification of the expression of CLECs between normal and tumor tissues was achieved using 110 normal controls selected from UCSC XENA.^2^ We applied the above data to generate ROC curves for evaluating the sensitivity and specificity of CLEC4s in HCC via qROC and ggplot2 package. Also, the mRNA expression level of CLEC4s was evaluated in the LIHC database from ONCOMINE.^3^ To validate the protein expression level of CLEC4s, immunohistochemical images of CLEC4s in HCC tissues and normal liver tissues were acquired from The Human Protein Atlas.^4^ The RNA expression data of the LIHC dataset based on GEPIA^5^ was evaluated to verify the mRNA expression level of CLEC4s in differential stages of HCC.

### Methylation Analysis

UALCAN (see text footnote 1), a comprehensive and user-friendly web resource for analyzing cancer data, is designed to perform pan-cancer gene expression analysis, identify biomarkers, evaluate gene expression in molecular subtypes of cancer, provide patient survival information, and other useful functions, helping researchers to gather valuable information about the genes/targets of interest. In this study, the promoter methylation level of CLEC4s in LIHC was explored via the UALCAN database (377 HCC samples and 50 normal samples). The statistical method selected was the Student’s *t*-test and *p* < 0.05 was judged to statistically significant.

### Relationship Between CLEC4s and Immune System

TIMER^6^ devotes to detecting the infiltration of immune cells in tumor tissue and analyzing its correlation with various cancers or gene expression. Besides, it also provided a quantitative analysis of the infiltrating proportion of six kinds of immune cells, including B cells, CD4^+^ T cells, CD8^+^ T cells, Neutrophils, Macrophages, and Dendritic cells. In this study, the correlation of immune infiltration (six kinds of immune cells) and immune checkpoints (PDCD1, LAG3, PDCD1LG2, and CD274) with mRNA expression of CLEC4s was analyzed on the TIMER website, respectively. In addition, the independent prognostic value of CLEC4S in HCC patients was also analyzed in the TIMER database. *P* < 0.05 was judged to statistically significant.

### Functional Enrichment Analysis

Metascape database^7^ is a powerful gene functional annotation analysis tool, which can be applied to protein annotation, enrichment analysis and construction of protein-protein interaction network. In this study, the enrichment analysis of CLEC4s was finished using different ontology categories of metascape database, including GO and KEGG pathway, DisGeNET and PaGenBase analysis. The analysis parameters are set as follows: Terms with a *p* < 0.01, a minimum count of 3, and an enrichment factor > 1.5. Besides, the Enrichr database^8^ provides not only usual enrichment analysis but also epigenetic modifications, transcription factor binding and expression in diseases and different cell types. The Enrichr database was used to predict transcription factors, miRNAs, target drugs and COVID19-related gene sets for CLEC4s. *P* < 0.05 was judged to statistically significant.

### Analysis of the Association of CLEC4s With Clinicopathological Features

The correlation between mRNA expressions of CLEC4s and clinicopathological features, including patient’s gender, age, smoking habit, individual cancer stage, TP53 mutation status, nodal metastasis status and tumor histology subtypes, were also conducted by UALCAN (see text footnote 1) database. The comparative method of the above research was student’s-test and *p* < 0.05 was judged to statistically significant.

### Survival Analysis

As integration of TCGA cancer data with GTEx normal tissue data, Gene Expression Profiling Interactive Analysis database (GEPIA)^9^ has been designed to reveal cancer subtypes, driver genes, differentially expressed or carcinogenic factors, excavating new cancer targets and markers. In this study, Overall survival (O.S.) and Disease-free survival (DFS) were analyzed using GEPIA (see text footnote 9).

### Cell Culture

The human liver cancer cell line HepG2 and human hepatic stellate cell line LX-2(normal liver cell line) were purchased from Procell Life Science and Technology Co., Ltd., with a STR identification certificate. These cells were cultured in Dulbecco’s modified Eagle’s medium (DMEM)-high glucose (Catalog: D6429, Sigma) medium containing 10% FBS (Catalog: 10099-141, Gibico) and 1% penicillin/streptomycin (Catalog: 15140-122,Gibico). All cells were cultured in a humidified incubator at 37°C and 5% CO_2_.

### qRT-PCR

According to the kit instructions, total RNA was extracted from LX-2 and HepG2 cells by RNA Easy Fast Tissue/Cell Kit (Catalog: DP451, Tiangen, Beijing, China). RNA quality was measured by SpectraMax^®^ QuickDrop^TM^ (Molecular Devices, Sunnyvale, CA, United States). Next, reverse transcription of 1 μg of total RNA through FastKing gDNA Dispelling RT SuperMix (Catalog: KR118-02, Tiangen, Beijing, China). The qRT-PCR reactions were prepared with Real Universal SYBR Green Premix (Catalog: FP201, Tiangen, Beijing, China) following the manufacturer’s instructions. Reactions were carried out and data analyzed in a LightCycler96 (Roche, Mannheim, Germany). We screened 18S rRNA from housekeeping genes GAPDH, B2M, and 18S rRNA that are more suitable for housekeeping genes. The 2^–ΔΔ^ ^Ct^ method was used to analyze the expression data of the target gene CLEC4s (CLEC4s expression normalized to the expression of 18S rRNA). The qPCR primers are shown in Supplementary Table 3. The experiment was repeated thrice, independently. We used GraphPad Prism 7 for graphing and statistics. The statistical method selected was the Student’s *t*-test.

## Results

### Down-Regulated CLECs in HCC Patients

**FIGURE 1:**
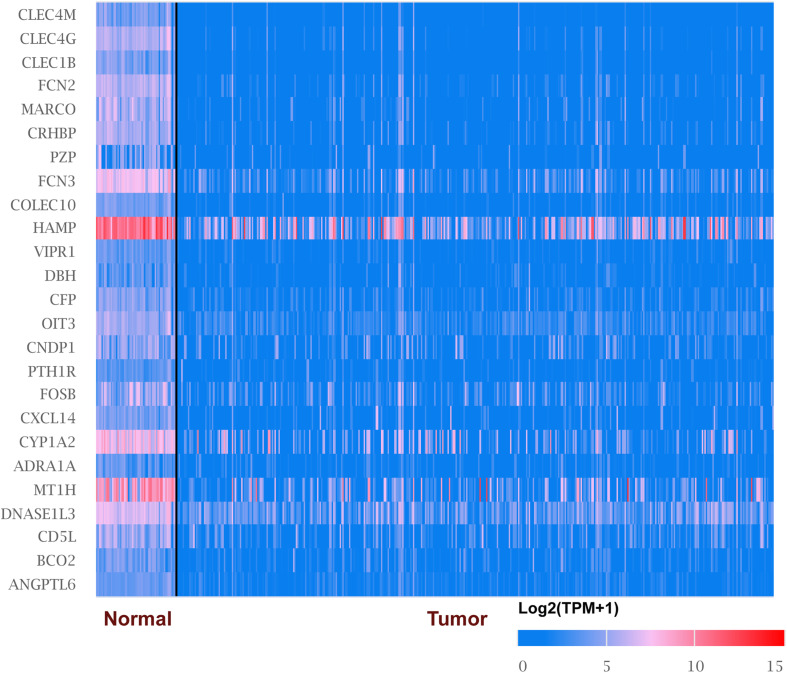
mRNA levels of top 25 under-expressed genes in liver hepatocellular carcinoma (LIHC) (ONCOMINE). The figure shows the numbers of datasets with statistically significant mRNA overexpression (red) or downregulated expression (blue) of genes in LIHC.

**FIGURE 2:**
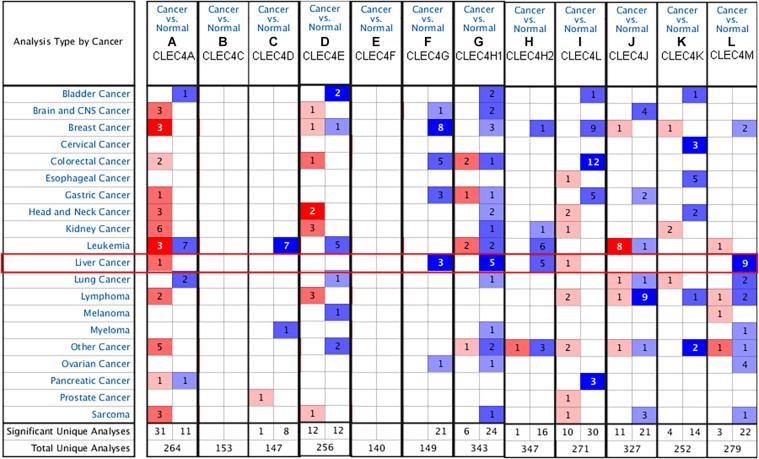
mRNA levels of CLEC4s in liver hepatocellular carcinoma (LIHC) (ONCOMINE). **(A)** CLEC4A; **(B)** CLEC4C; **(C)** CLEC4D; **(D)** CLEC4E; **(E)** CLEC4F; **(F)** CLEC4G; **(G)** CLEC4H1; **(H)** CLEC4H2; **(I)** CLEC4J; **(J)** CLEC4K; **(K)** CLEC4L; **(L)** CLEC4M. The figure shows the numbers of datasets with statistically significant mRNA overexpression (red) or downregulated expression (blue) of genes in LIHC.

**FIGURE 3:**
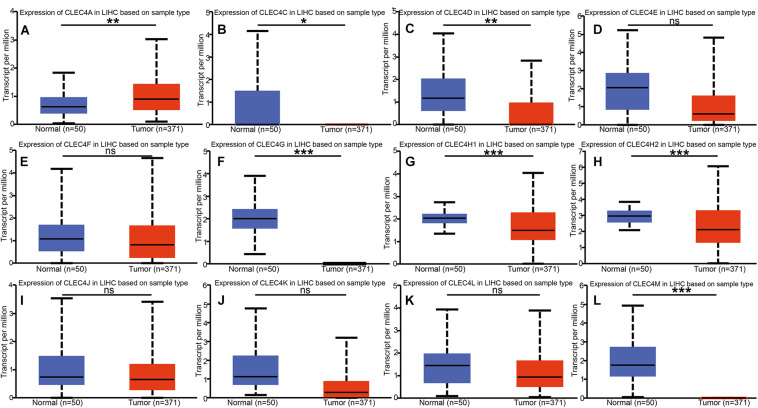
mRNA expression of distinct CLEC4s in HCC tissues and adjacent normal liver tissues (UALCAN). mRNA expressions of 11 CLEC4s were found to be lower-expressed in primary HCC tissues compared to normal controls except CLEC4A. **(A)** CLEC4A; **(B)** CLEC4C; **(C)** CLEC4D; **(D)** CLEC4E; **(E)** CLEC4F; **(F)** CLEC4G; **(G)** CLEC4H1; **(H)** CLEC4H2; **(I)** CLEC4J; **(J)** CLEC4K; **(K)** CLEC4L; **(L)** CLEC4M. *p < 0.05; **p < 0.01; ***p < 0.001; ns, not significant.

We explored the expression level of CLECs between HCC and normal tissues based on the UALCAN database. Lower CLECs levels were expressed in HCC tissues compared to normal liver tissues (Figure 1). Of the top 250 down-regulated CLECs, CLEC4M and CLEC4G were the topmost. As a result, we chose the CLEC4s family for further analysis. The transcriptional levels of CLEC4s were compared in 20 cancer types to normal tissues based on data from ONCOMINE (Figure 2). We found significantly higher mRNA levels of CLEC4A and CLEC4L in HCC tissues compared to normal liver tissues (Figure 2 and Table 1), whereas the expression of CLEC4G/H1/H2/M was significantly lower in liver carcinoma (Table 1) (Chen et al., 2002; Wurmbach et al., 2007; Mas et al., 2009; Roessler et al., 2010). To validate the mRNA expression level of CLEC4s in liver tissues, the transcriptional level of CLEC4s was analyzed in the TCGA database (Figures 3A–L). The expression level of CLEC4s, including CLEC4C (*P* < 0.05), CLEC4D (*P* < 0.05), CLEC4E (*P* < 0.05), CLEC4J (*P* < 0.05), CLEC4K (*P* < 0.05), CLEC4G (*P* < 0.05), CLEC4H (*P* < 0.05), CLEC4M (*P* < 0.05), were significantly lower in HCC samples compared to normal hepatic samples, whereas the expression of CLEC4A was higher in HCC (Figures 3A–L). Further analysis of the expression level of CLEC4s between unpaired tumor samples and normal controls revealed significantly low CLEC4A/D/E/G/H1/J/M levels expressed in HCC samples (Supplementary Figures 1A,B). The comparison between paired HCC tissues and adjacent tissues demonstrated significantly lower CLEC4C/D/E/F/G/H1/H2/K/M levels expressed in HCC samples, except for CLEC4A/J/L (Supplementary Figures 1C,D).

**TABLE 1:** The mRNA levels of CLEC4s family in different types of LIHC tissues and normal liver tissues at transcriptional level (ONCOMINE).

**Gene**	**Type**	**Fold change**	***P*-value**	**References**
CLEC4A	Hepatocellular Carcinoma	1.656	1.41E-09	Mas et al., 2009
CLEC4G	Hepatocellular Carcinoma	–33.571	2.23E-21	Wurmbach et al., 2007
	Liver Cell Dysplasia	–2.016	3.64E-05	Wurmbach et al., 2007
	Cirrhosis	–1.77	3.70E-04	Wurmbach et al., 2007
CLEC4H1	Cirrhosis	–2.027	1.39E-14	Mas et al., 2009
	Hepatocellular Carcinoma	–2.099	1.33E-10	Mas et al., 2009
	Hepatocellular Carcinoma	–1.992	5.73E-14	Chen et al., 2002
	Hepatocellular Carcinoma	–2.022	3.55E-32	Roessler et al., 2010
	Hepatocellular Carcinoma	–1.545	3.14E-05	Wurmbach et al., 2007
CLEC4H2	Hepatocellular Carcinoma	–1.94	1.12E-13	Chen et al., 2002
	Hepatocellular Carcinoma	–1.922	1.45E-29	Roessler et al., 2010
	Hepatocellular Carcinoma	–2.319	2.68E-05	Roessler et al., 2010
	Hepatocellular Carcinoma	–1.671	1.47E-06	Mas et al., 2009
	Hepatocellular Carcinoma	–1.563	5.16E-04	Wurmbach et al., 2007
CLEC4L	Hepatocellular Carcinoma	1.645	2.70E-02	Chen et al., 2002
CLEC4M	Hepatocellular Carcinoma	–28.107	9.06E-57	Chen et al., 2002
	Focal Nodular Hyperplasia of the Liver	–4.298	6.00E-03	Chen et al., 2002
	Hepatocellular Adenoma	–20.529	4.60E-02	Chen et al., 2002
	Hepatocellular Carcinoma	–9.276	1.49E-18	Roessler et al., 2010
	Hepatocellular Carcinoma	–36.431	2.77E-14	Wurmbach et al., 2007
	Liver Cell Dysplasia	–3.836	2.61E-06	Wurmbach et al., 2007
	Cirrhosis	–2.965	5.43E-05	Wurmbach et al., 2007
	Hepatocellular Carcinoma	–4.361	3.28E-94	Roessler et al., 2010
	Hepatocellular Carcinoma	–2.014	3.93E-06	Mas et al., 2009

**FIGURE 4:**
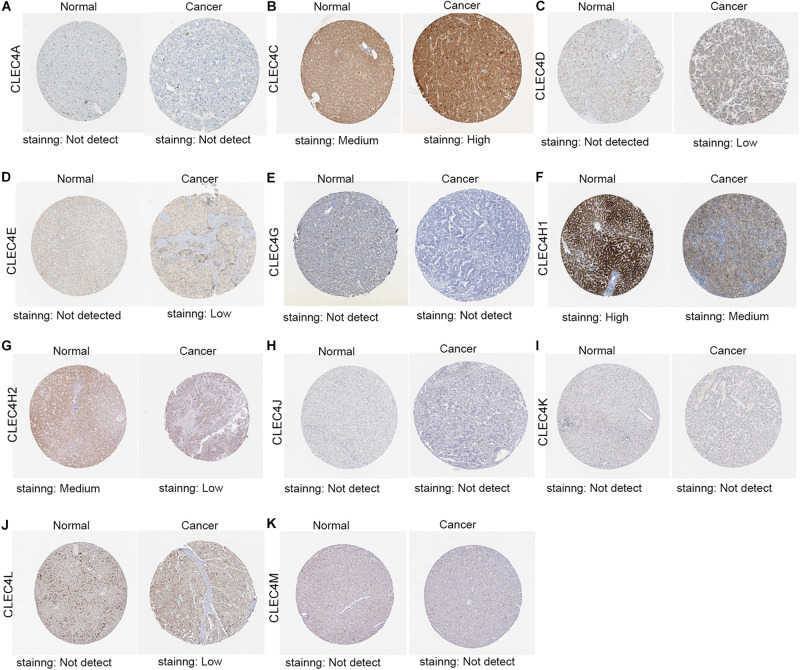
Representative immunohistochemistry images of CLEC4s in HCC tissues and normal liver tissues (Human Protein Atlas). The IHC results showed CLEC4A/G/J/K/M was not detected both in normal and liver cancer tissues **(A,E,H,I,K)**, while CLEC4C was highly expressed in HCC tissues **(B)**, CLEC4H1/H2 was lower expressed in HCC tissues compared to normal tissues (Figures 4F,G) and CLEC4D/E/L was observed low expression in HCC tissues, but not detected in normal tissues **(C,D,J)**.

We also tested the expression levels of CLEC4s in HCC at the protein level through the Human Protein Atlas (Figures 4A–K). The IHC results revealed no CLEC4A/G/J/K/M in normal and liver cancer tissues (Figures 4A,E,H,I,K), whereas CLEC4C was highly expressed in HCC tissues (Figure 4B), and CLEC4H1/H2 was lower expressed in HCC tissues compared to normal tissues(Figures 4F,G). Also, CLEC4D/E/L expression in HCC tissues and normal tissues was low and absent, respectively (Figures 4C,D,J).

### The Association of CLEC4s With Clinicopathological Features of HCC

**FIGURE 5:**
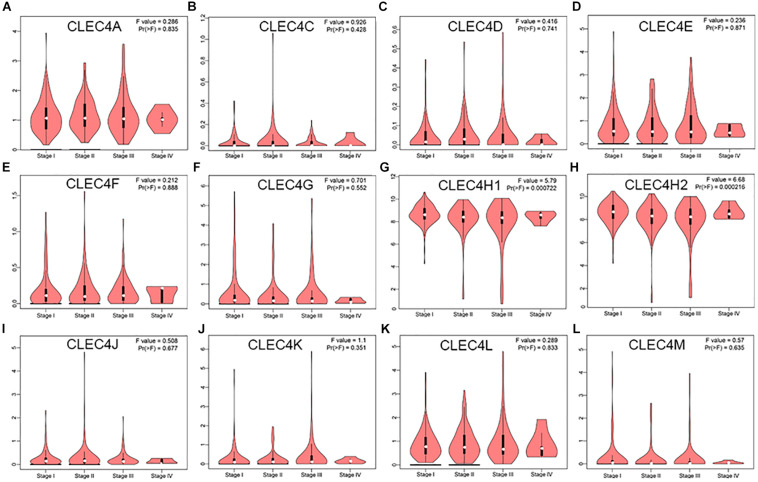
The correlation between CLEC4s with stage of HCC patients (GEPIA). **(A)** CLEC4A; **(B)** CLEC4C; **(C)** CLEC4D; **(D)** CLEC4E; **(E)** CLEC4F; **(F)** CLEC4G; **(G)** CLEC4H1; **(H)** CLEC4H2; **(I)** CLEC4J; **(J)** CLEC4K; **(K)** CLEC4L; **(L)** CLEC4M.

By comparing the expression levels of CLEC4s based on individual cancer stages and tumor grades, the expression of CLEC4A/C/D/E/F/G/J/K/L/M showed no significant relation with clinical stages based on GEPIA database (Figures 5A–F, I–L), while CLEC4H1 (ASGR1, *P* = 0.000) and CLEC4H2(ASGR2, *P* = 0.000) was significantly related to clinical stages based on the GEPIA database (Figures 5G,H). To verify these results, we explored the association of CLEC4s with individual cancer stages from UALCAN database and found that CLEC4s were associated with individual cancer stages (Supplementary Figure 2) and tumor grade (Supplementary Figure 3). Besides, CLEC4D/E/G/H1/H2/J/K/M were significantly correlated with individual cancer stages (Supplementary Figures 2A–L). Further exploration of the relationship between CLEC4s and tumor grade revealed that a significant association of the expression of CLEC4A/F/G/H1/H2/M with tumor grade (Supplementary Figure 3F–H,L). Collectively, these findings demonstrated a remarkable role of CLEC4s in hepatocarcinogenesis.

### The Prognostic Value of CLEC4 Members in HCC Patients

After establishing the association of CLEC4s expression with HCC outcome via the GEPIA database, we found that patients expressing higher CLEC4H1 (ASGR1, *P* = 0.013) and CLEC4H2 (ASGR2, *P* = 0.018) levels were characterized by longer overall survival compared to patients exhibiting lower expression (Supplementary Figures 4A–K). Also, HCC patients expressing higher CLEC4H1 (ASGR1, *P* = 0.018) levels exhibited a longer disease-free survival rate (Supplementary Figures 5A–K). Evaluation of the specificity of CLEC4s in HCC with ROC curves revealed high specificity of CLEC4s in HCC (Supplementary Figures 6A–C). Notably, CLEC4G demonstrated the highest specificity in predicting the prognosis (AUC = 0.970).

### Down-Regulated Promotor Methylation Levels of CLEC4s in LIHC

**FIGURE 6:**
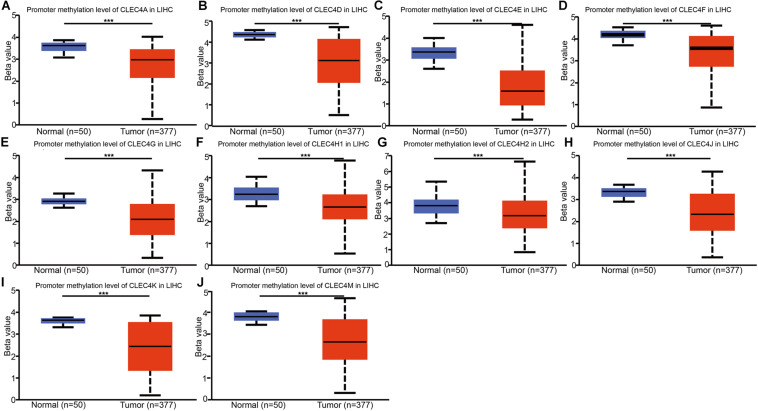
Promotor methylation levels of CLEC4s in HCC tissues and adjacent normal liver samples. Promoter methylation expression levels of 10 CLEC4s were found to be downregulated in primary HCC tissues compared to normal controls. **(A)** CLEC4A; **(B)** CLEC4D; **(C)** CLEC4E; **(D)** CLEC4F; **(E)** CLEC4G; **(F)** CLEC4H1; **(G)** CLEC4H2; **(H)** CLEC4J; **(I)** CLEC4K; **(J)** CLEC4M. ***p < 0.001.

We explored the potential mechanisms of the effect of CLEC4s in HCC through the analysis of the promoter methylation levels of CLEC4s in LIHC from UALCAN dataset. The results showed that CLEC4s, including CLEC4A (*P* < 0.05), CLEC4D (*P* < 0.05), CLEC4E (*P* < 0.05), CLEC4J (FCER2, *P* < 0.05), CLEC4K (CD207, *P* < 0.05), CLEC4G (*P* < 0.05), CLEC4H1 (ASGR1, *P* < 0.05), CLEC4M (*P* < 0.05) CLEC4H2 (ASGR2, *P* < 0.05) exhibited significantly low promotor methylation levels (Figures 6A–J).

### Immune Cell Infiltration of CLEC4s in HCC Patients

**FIGURE 7:**
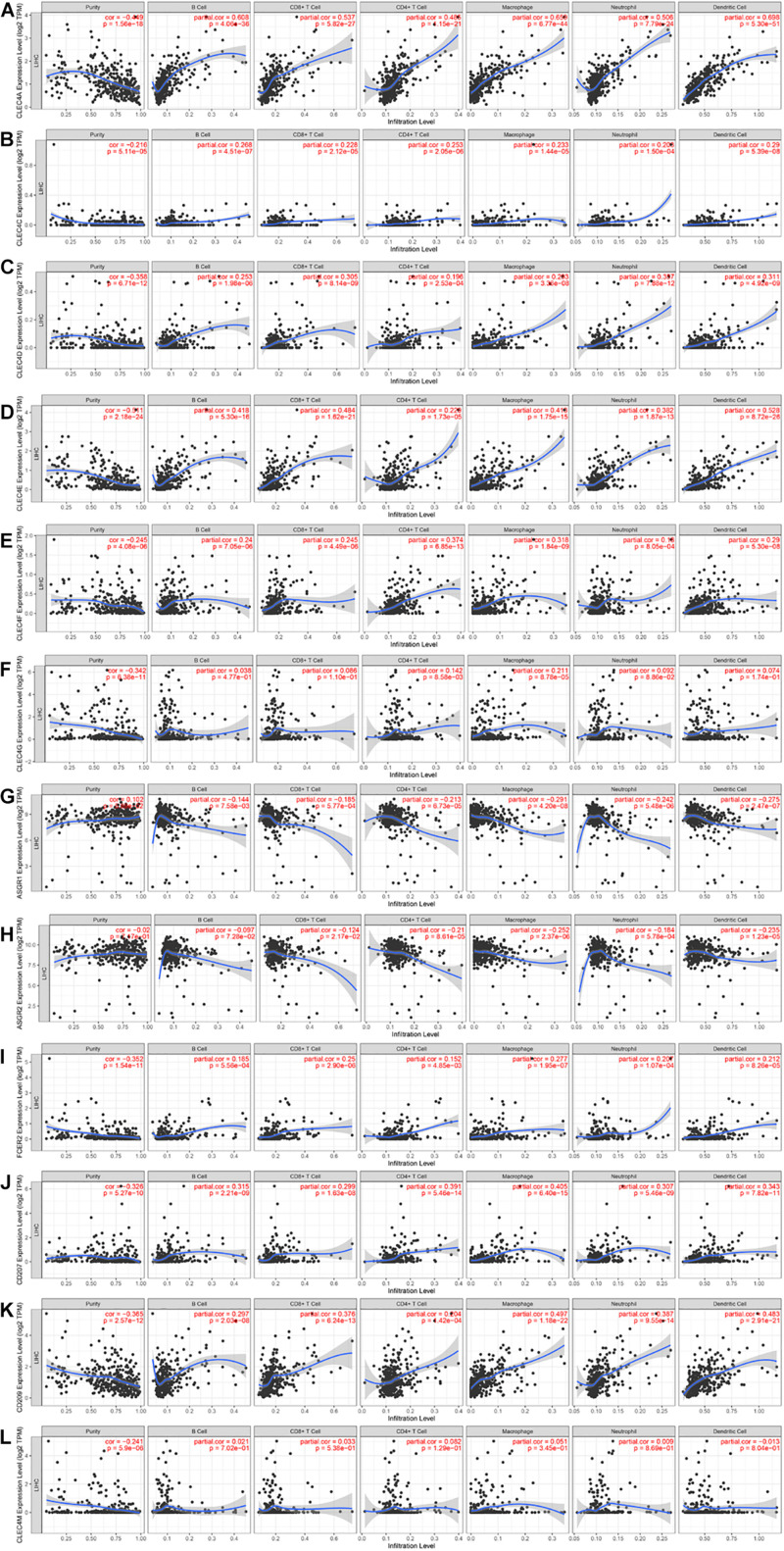
The correlation between CLEC4s and immune cell infiltration (TIMER). The correlation between the abundance of immune cell and the expression of **(A)** CLEC4A, **(B)** CLEC4C, **(C)** CLEC4D, **(D)** CLEC4E, **(E)** CLEC4F, **(F)** CLEC4G, **(G)** CLEC4H1, **(H)** CLEC4H2, **(I)** CLEC4J, **(J)** CLEC4K, **(K)** CLEC4L, and **(L)** CLEC4M in HCC.

**FIGURE 8:**
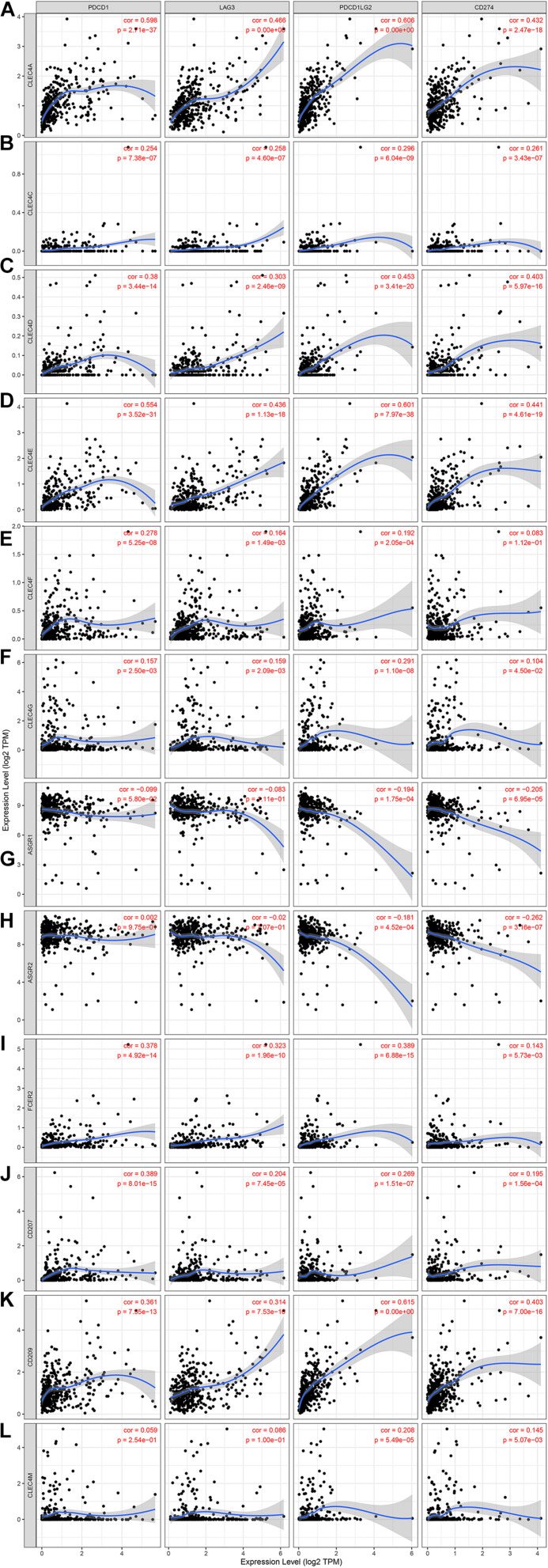
The correlation between different expressed CLEC4s and PDCD1/LAG3/PDCD1LG2/CD274 (TIMER). The correlation between the abundance of immune cell and the expression of **(A)** CLEC4A, **(B)** CLEC4C, **(C)** CLEC4D, **(D)** CLEC4E, **(E)** CLEC4F, **(F)** CLEC4G, **(G)** CLEC4H1, **(H)** CLEC4H2, **(I)** CLEC4J, **(J)** CLEC4K, **(K)** CLEC4L, and **(L)** CLEC4M in HCC.

Because CLEC4s are crucial candidates for the immune response, we investigated the correlation between CLEC4s and immune cell infiltration via the Timer database (see Figure 7 and Tables 2, 3). The expression levels of CLEC4A, CLEC4C, CLEC4D, CLEC4E, and CLEC4F were all positively correlated with the infiltration of B cells, CD8^+^ T cells, CD4^+^ T cells, macrophages, neutrophils, and dendritic cells (Figures 7A–E, *P* < 0.05). The expression of CLEC4G was positively correlated with CD4^+^ T cells and macrophages (Figure 7F, *P* < 0.05). The expression levels of CLEC4H1 (ASGR1) and CLEC4H2 (ASGR2) exhibited a negative correlation with B cells, CD8^+^ T cells, CD4^+^ T cells, macrophages, neutrophils, and dendritic cells (Figures 7G,H, *P* < 0.05). The expression levels of CLEC4J (FCER2), CLEC4K (CD207), CLEC4L (CD209), were positively correlated with B cells, CD8^+^ T cells, CD4^+^ T cells, macrophages, neutrophils, and dendritic cells (Figures 7I–K, *P* < 0.05). CLEC4M expression was positively correlated with B cells, CD8^+^ T cells, CD4^+^ T cells, macrophages, neutrophils, and dendritic cells (Figure 7L, *P* < 0.05). Additionally, CLEC4s, except for CELC4H1/2, exhibited a significant positive associated with Programmed cell death protein 1 (PDCD-1), Lymphocyte-activation gene 3 (LAG3), and Programmed Cell Death 1 Ligand 2 (PDCD1LG2) (Figures 8A–L).

**TABLE 2:** The cox proportional hazard model of CLEC4s and six tumor-infiltrating immune cells in LIHC (TIMER).

**Cancer**	**Variable**	***P***
LIHC	B cell	0.488703
LIHC	CD8^+^ T cell	0.550693
LIHC	CD4^+^ T cell	0.453671
LIHC	Macrophage	0.152763
LIHC	Neutrophil	0.200464
LIHC	Dendritic Cell	0.288267
LIHC	CLEC4A	0.257092
LIHC	CLEC4C	0.890902
LIHC	CLEC4D	0.130274
LIHC	CLEC4E	0.234832
LIHC	CLEC4F	0.404576
LIHC	CLEC4G	0.735808
LIHC	ASGR1	0.014147
LIHC	ASGR2	0.020965
LIHC	FCER2	0.582567
LIHC	CD207	0.809007
LIHC	CD209	0.206008
LIHC	CLEC4M	0.755055

**TABLE 3:** Multivariate analysis of overall survival in 315 LIHC patients.

	**Coef**	**HR**	**95%CI_l**	**95%CI_u**	***p*-value**	**Sig**
Age	0.016	1.016	0.999	1.034000e + 00	0.069	^■^
Gendermale	0.052	1.053	0.669	1.659000e + 00	0.823	
Stage2	0.246	1.278	0.747	2.186000e + 00	0.370	
Stage3	0.797	2.220	1.358	3.628000e + 00	0.001	**
Stage4	1.399	4.050	1.088	1.507300e + 01	0.037	*
Purity	1.060	2.886	0.866	9.619000e + 00	0.084	^■^
B_cell	–8.621	0.000	0.000	5.150000e-01	0.034	*
CD8_Tcell	–7.538	0.001	0.000	1.420000e-01	0.008	**
CD4_Tcell	–10.041	0.000	0.000	2.820000e-01	0.025	*
Macrophage	14.026	1234392.939	744.103	2.047734e + 09	0.000	***
Neutrophil	–3.010	0.049	0.000	6.370710e + 04	0.675	
Dendritic	5.785	325.352	3.888	2.722593e + 04	0.010	*
CLEC4A	–0.158	0.854	0.380	0.380	1.916000e + 00	
CLEC4C	2.644	14.069	0.054	3.680792e + 03	0.352	
CLEC4D	–1.122	0.326	0.005	2.095100e + 01	0.598	
CLEC4E	0.295	1.343	0.663	2.721000e + 00	0.414	
CLEC4F	–0.409	0.664	0.208	2.120000e	0.490	
CLEC4G	–0.593	0.553	0.300	1.017000e + 00	0.057	^■^
ASGR1	0.379	1.461	1.063	2.007000e + 00	0.019	*
ASGR2	–0.241	0.786	0.786	0.569	1.085000e + 00	
FCER2	0.836	2.307	1.097	4.849000e + 00	0.027	*
CD207	0.253	1.287	0.955	1.736000e + 00	0.098	^■^
CD209	–0.048	0.953	0.632	1.436000e + 00	0.817	

***p < 0.001; ** p < 0.01;* p < 0.05; ■ 0.05 < p < 0.1.

**FIGURE 9:**
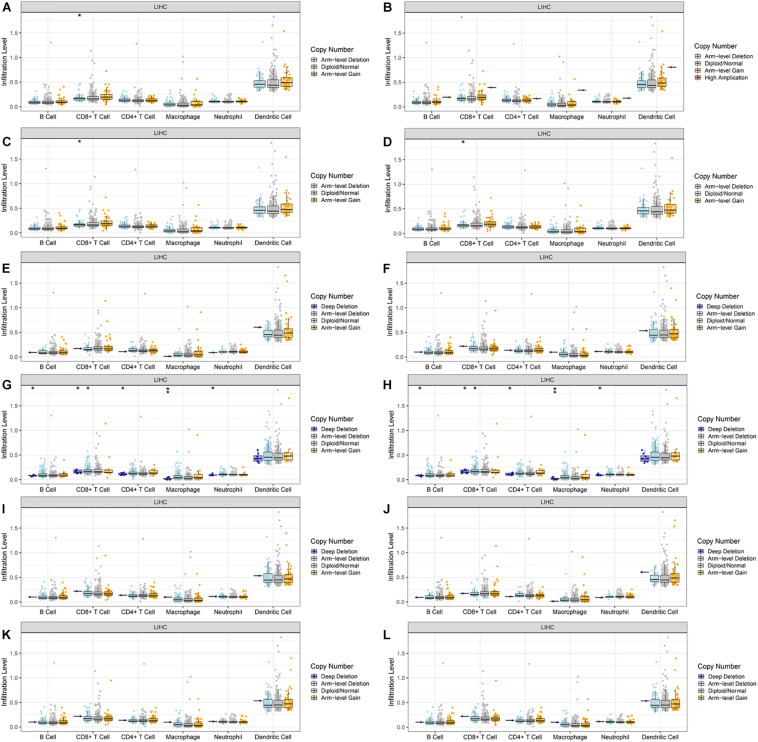
The connection between the copy number variation (CNV) of CLEC4s and the immune cell infiltration level in HCC. **(A)** CLEC4A, **(B)** CLEC4C, **(C)** CLEC4D, **(D)** CLEC4E, **(E)** CLEC4F, **(F)** CLEC4G, **(G)** CLEC4H1, **(H)** CLEC4H2, **(I)** CLEC4J, **(J)** CLEC4K, **(K)** CLEC4L, and **(L)** CLEC4M. **p < 0.01; *p < 0.05.

Lastly, we explored the correlation between the copy number variation (CNV) of CLEC4s and the immune cell infiltration level (Figures 9A–L). The results demonstrated that CLEC4A/D/E CNV was correlated with CD8^+^ T cell infiltration (*P* < 0.05) (Figures 9A,C,D). CLEC4H1/2 CNV was correlated with B cells, CD8^+^ T cells, CD4^+^ T cells, macrophages, and neutrophils (Figures 9G,H, *P* < 0.05). These results implicated the potential role of CLEC4s in the development of HCC through immune response regulation.

### Functional Enrichment, Co-expression, and Interaction Analyses of CLEC4s in HCC Patients

**FIGURE 10:**
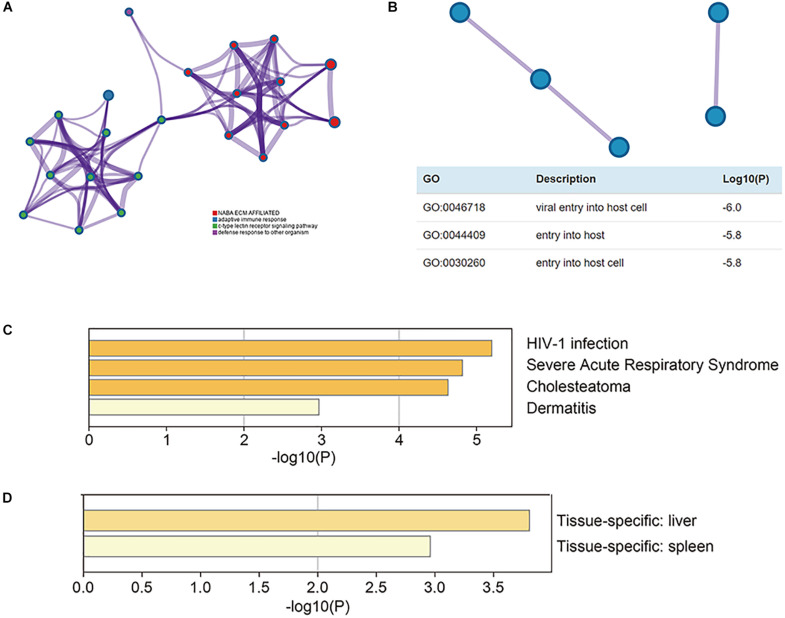
The functional enrichment analysis of CLEC4s. The association of CLEC4s with their neighboring genes analyzed with Cytoscape was shown in **(A)** and protein-protein interaction evaluated with MCODE was shown in **(B)**. CLECL4 was involved in HIV-1 infection, severe acute respiratory syndrome, cholesteatoma, and dermatitis **(C)**. CLEC4s were specific in liver and spleen tissue **(D)**.

The functional enrichment analysis of CLEC4s was performed through the Metascape website. To understand the relationship between CLEC4s and LIHC, we performed PPI (protein-protein interaction) network and mCODE components analysis (Figures 10A,B). Enrichment analysis in DisGeNET showed that CLECL4 mediated HIV-1 infection, severe acute respiratory syndrome, cholesteatoma, and dermatitis (Figure 10C); thus, we confirmed its association with virus infection. More interestingly, enrichment analysis in PaGenBase showed the specificity of CLEC4s in liver and spleen tissue (Figure 10D).

### Transcription Factor Targets, COVID-19 Related Gene Sets, miRNA Targets, and Drug Targets of CLEC4s in HCC Patients

We predicted the potential mechanisms through which CLEC4s induces the progression of HCC by exploring the possible transcription factor targets of CLEC4s in the Enrichr database. Four transcription factor targets (NFAT2, STAT1, STAT5B, and POU2F1) were found (Table 4). Based on Enrichr, we found two possible miRNA targets, including hsa-miR-4278 and hsa-miR-324-5p (Table 5). Because the viral entry into host cells is associated with CLEC4s, we further evaluated the association of CLEC4s with COVID 19 based on COVID 19 drug and gene sets library; Results demonstrated the potential role of CLEC4s in COVID 19 infection (Supplementary Table 1). Additional findings on drugs that target CLEC4s provide insights into the management of COVID 19 infection (Supplementary Table 2). Next, we analyzed the correlation analysis between CLEC4s and liver cancer drug targets at the mRNA level (Supplementary Figure 7 and Supplementary Table 4). The results found that the CLEC4s are significantly correlated with FDA-approved live targets for liver cancer (VEGFR, FGFR, PDGFR, etc.).

**TABLE 4:** The potential transcript factors of CLEC4s based on Enrichr.

**Term**	***P*-value**	**Odds Ratio**	**Combined Score**
NFAT2 (human)	0.005272	3.179506	16.67748
STAT1 (mouse)	0.00529	18.38235	96.36052
STAT5B (human)	0.005335	3.172589	16.60349
POU2F1 (human)	0.021826	2.680486	10.25192
LOC135440 (human)	0.059128	5.060729	14.312

**TABLE 5:** The potential miRNA targets of CLEC4s based on Enrichr.

**Term**	***P*-value**	**Odds ratio**	**Combined score**
hsa-miR-4278	0.013055	2.68076	11.63068
hsa-miR-324-5p	0.032615	2.229867	7.63281
mmu-miR-154	0.053274	2.409406	7.065101
hsa-miR-4529-3p	0.061481	2.094972	5.842942
hsa-miR-3689b	0.072227	2.010724	5.284052
hsa-miR-3689a-5p	0.072227	2.010724	5.284052
hsa-miR-3689e	0.072227	2.010724	5.284052
hsa-miR-3689f	0.072227	2.010724	5.284052
hsa-miR-1468	0.075466	3.029079	7.827353
hsa-miR-196a	0.108323	1.953125	4.341083

### Experimental Verification and Clinical Correlation Analysis of CLEC4s

We assessed mRNA expression levels in HepG2 and LX-2 cell lines via qPCR. Except for CLEC4H1/H2, CLEC4s are significantly low expressed in HepG2. CLEC4H1/H2 was significantly up-regulated in HepG2 (Supplementary Figures 8A–M, *p* < 0.05). In addition, we downloaded the RNA-seq expression data of CLEC4s in liver cancer cells in the CCLE^10^ database. We used the bininformatics^11^ database to draw a clustering heat map of CLEC4s families to verify our experimental results (Supplementary Figure 8N and Supplementary Table 5). CLEC4H1/H2 was highly expressed, and other genes are expressed at low levels in liver cancer cell lines. Clinical and gene expression data of 374 primary tumors and 50 paracancerous samples were downloaded from TCGA, including T, N, M, pathologic stage, gender, age, histological grade, and OS event (Supplementary Tables 6–17). The expression level of CLEC4D was significantly correlated with N stage. CLEC4F was significantly correlated with gender. CLEC4G was significantly correlated with histologic grade. ASGR1 (CLEC4H1) was significantly correlated with T stage and histologic grade. ASGR2 (CLEC4H2) was significantly correlated with T stage and gender. FCER2 (CLEC4J) was significantly correlated with age. CD209 (CLEC4L) was significantly correlated with OS event.

## Discussion

CLEC4s, a member of the C-type lectin receptors, are among the constituents of the immune system. Currently, studies have reported the association of aberrant immune response with the development of HCC (Wu et al., 2020); however, the relationship between CLEC4s and HCC is still elusive.

In the present study, by exploring the expression and biological function of CLEC4s in HCC, we revealed that the expression levels ofCLEC4s are low in HCC and are related to clinical and pathological stages of HCC. Among them, CLEC4H1/2 is related to a good prognosis, implicating the potentially critical role of CLEC4s in HCC. Through ROC, we verified the diagnosis value of CLEC4 in HCC and analyzed its promoter methylation level to lay a foundation for elucidating its mechanism in HCC. Of note, low levels of CLEC4s methylation were reported, suggesting that epigenetic modification of CLEC4s may be vital in the development of HCC. Further analysis of the potential transcription factors and miRNA targets revealed 10 related transcription factors (ETV4, Sox17, ZFHX3, TEAD1, EIF4EBP1, FOX11, PITX2, HNF4A, and PITX1) and two miRNA targets (hsa-miR-4278 and hsa-miR-324-5p). In particular, ETV4 was one of the transcript targets, previously reported to participate in the regulation proliferation and metastasis of thyroid cancer (Yu et al., 2020). SOX17 transcript has also been proven to play a crucial role in the progression of various cancers, including lung cancer, hepatic carcinoma, and gastric cancer (Olbromski et al., 2020). Zinc finger homeobox 3 (ZFHX3) is essential to the tumorigenesis of HCC-dependent angiogenesis (Fu et al., 2020). Moreover, HNF4α was reported to induce liver cancer cell proliferation and migration (Xu Q. et al., 2020), whereas pituitary homeobox 1 (PITX1) exerted tumor suppressor effects in HCC (Tai et al., 2016). Interestingly, these transcript targets could interact with each other in HCC. A recent study found a direct interaction of HNF4α with TEA domain family members (TEAD), consequently blocking the transcriptional activity of Yes-associated protein (YAP1)/TEAD in rats with HCC (Cai et al., 2017). These findings validate the potential interaction between CLEC4s and the above transcripts, which is crucial in the progression of HCC.

Based on the analysis of the relationship between CLEC4s and immune cell infiltration in HCC, we found a significant correlation of CLEC4s with B cells, CD4^+^ T cells, N.K cells, CD8^+^ T cells, and D.C. cells. These observations are suggestive of the critical roles that CLEC4s may play in the occurrence of HCC, particularly via immune response regulation. Furthermore, the significant correlation between the expression of CLEC4s and the immune set point suggests their potential roles in immunotherapy. Finally, through functional enrichment analysis, we demonstrated the association of CLEC4 with the adaptive immune response and in the virus infection process. Also, we found a correlation between CLEC4s and COVID 19 infection, which concur with the previous findings by Holter et al. (2020). The identified drugs that target CLEC4s also provide possible management options for COVID 19 infection and offer a basis for CLEC4s in immune regulation.

## Conclusion

The present study explored the role of CLEC4s in immune response and HCC development. We found that CLEC4s play a key role in the development of HCC and are associated with various immune cell infiltration. These findings provide a theoretical basis for further elucidation of the pathogenesis of liver cancer and present a new target and direction for clinical treatment. However, the analyses were based on online databases. Therefore, further studies integrating animal and clinical experiments are warranted to validate the findings and promote the clinical application of CLEC4s in HCC management.

Our results provide evidence for new immuno- therapy targets in HCC.

## Data Availability Statement

The datasets presented in this study can be found in online repositories. The names of the repository/repositories and accession number(s) can be found in the article/Supplementary Material.

## Author Contributions

YZ, HW, LF, BL, and ZP designed this research. YZ and HW performed the analyses and prepared the manuscript. YZ, HW, MF, and XH performed the literature search and the initial analyses. YZ, HW, BL, and ZP performed analyses and data interpretation. HW, LF, and MF edited the manuscript. All authors have read and agreed to the published version of the manuscript.

## Conflict of Interest

The authors declare that the research was conducted in the absence of any commercial or financial relationships that could be construed as a potential conflict of interest.

## Publisher’s Note

All claims expressed in this article are solely those of the authors and do not necessarily represent those of their affiliated organizations, or those of the publisher, the editors and the reviewers. Any product that may be evaluated in this article, or claim that may be made by its manufacturer, is not guaranteed or endorsed by the publisher.

## Footnotes

1
http://ualcan.path.uab.edu

2
https://xenabrowser.net/datapages/

3
www.oncomine.org

4
https://www.proteinatlas.org

5
http://gepia.cancer-pku.cn/index.html

6
https://cistrome.shinyapps.io/timer/

7
http://metascape.org/

8
https://maayanlab.cloud/Enrichr/

9
http://gepia.cancer-pku.cn

10
https://portals.broadinstitute.org/ccle

11
http://www.bioinformatics.com.cn

## Supplementary Material

Supplementary Figure 1 mRNA expression level of CLEC4s in HCC compared to normal control. **(A,B)** Non-paired tissues, **(C,D)** paired tissues. ^∗^*p*< 0.05; ^∗∗^*p*< 0.01; ^∗∗∗^*p* < 0.001; ns, not significant.

Supplementary Figure 2 Relationship between mRNA expression of CLEC4s and individual cancer stages of HCC patients. mRNA expressions of CLEC4s were remarkably down-regulated in HCC patients except CLEC4A. **(A)** CLEC4A, **(B)** CLEC4C, **(C)** CLEC4D, **(D)** CLEC4E, **(E)** CLEC4F, **(F)** CLEC4G, **(G)** CLEC4H1, **(H)** CLEC4H2, **(I)** CLEC4J, **(J)** CLEC4K, **(K)** CLEC4L, and **(L)** CLEC4M. ^∗^*p* < 0.05; ^∗∗^*p*< 0.01; ^∗∗∗^*p* < 0.001; ns, not significant.

Supplementary Figure 3 Association of mRNA expression of CLEC4s with tumor grades of HCC patients. **(A)** CLEC4A, **(B)** CLEC4C, **(C)** CLEC4D, **(D)** CLEC4E, **(E)** CLEC4F, **(F)** CLEC4G, **(G)** CLEC4H1, **(H)** CLEC4H2, **(I)** CLEC4J, **(J)** CLEC4K, **(K)** CLEC4L, and **(L)** CLEC4M. ^∗^*p* < 0.05; ^∗∗^*p* < 0.01; ^∗∗∗^*p* < 0.001; ns, not significant.

Supplementary Figure 4 Prognostic value of mRNA expression of CLEC4s in HCC patients (GEPIA). Higher mRNA expressions of CLEC4H1 and CLEC4H2 were significantly associated with longer overall survival of cancer patients **(F,G)**. **(A)** CLEC4A, **(B)** CLEC4D, **(C)** CLEC4E, **(D)** CLEC4F, **(E)** CLEC4E, **(F)** CLEC4H1, **(G)** CLEC4H2, **(H)** CLEC4J, **(I)** CLEC4K, **(J)** CLEC4L, **(K)** CLEC4M.

Supplementary Figure 5 Disease-free survival value of mRNA expression of CLEC4s in HCC patients (GEPIA). Generally, CLEC4s mRNA expression showed no correlation with disease-free survival rate in HCC. **(A)** CLEC4A, **(B)** CLEC4D, **(C)** CLEC4E, **(D)** CLEC4F, **(E)** CLEC4E, **(F)** CLEC4H1, **(G)** CLEC4H2, **(H)** CLEC4J, **(I)** CLEC4K, **(J)** CLEC4L, **(K)** CLEC4M.

Supplementary Figure 6 The sensitivity and specificity of CLEC4s in HCC. The diagnostic value of CLEC4A/C/D/E **(A)**, CLEC4F/G/H1/H2 **(B)**, and CLEC4J/L/K/M **(C)** in HCC.

Supplementary Figure 7 The correlation between CLEC4s and liver cancer drug targets at mRNA level. ASGR1(CLEC4H1), ASGR2(CLEC4H2), FCER2(CLEC4J), CD207(CLEC4K), CD209(CLEC4L), VEGFR1/2/3(FLT1/KDR/FLT4), PDGFR-α/β (PDGFRA/PDGFRB). ^∗^*p* < 0.05; ^∗∗^*p* < 0.01; ns, not significant.

Supplementary Figure 8 Analysis of CLEC4 expression in liver cancer cell lines. **(A–M)** qPCR detected the expression level of CLEC4s in HepG2;(N)Analyze the expression level of CLEC4s in liver cancer cell lines in the CCLE database. ^∗^*p*< 0.05; ^∗∗^*p* < 0.01; ^∗∗∗^*p* < 0.001; ns, not significant.

Supplementary Figure 9 Schematic representation of the overall workflow of this research. The flow chart summarized the overall steps for the target gene identification and analysis.

Supplementary Table 1 The potential COVID-19 related gene sets associated with CLEC4.

Supplementary Table 2 The predicted drugs targeting CLEC4s for treating COVID-19 based on Drug matrix.

Supplementary Table 3 qPCR primers used in this study.

Supplementary Table 4 The correlation between CLEC4s and liver cancer drug targets.

Supplementary Table 5 The expression level of CLEC4s in hepatocarcinoma cell lines.

Supplementary Table 6 Characteristics of patients with HCC based on TCGA.

Supplementary Tables 7–17 Relationship between CLEC4s mRNA expression and clinical characteristics in HCC.
