# Supplementary figures and images for: CLEC4s as Potential Therapeutic Targets in Hepatocellular Carcinoma Microenvironment
Source: Front Cell Dev Biol. 2021 Aug 2;9:681372. doi: 10.3389/fcell.2021.681372 (PMC8367378; doi:10.3389/fcell.2021.681372)

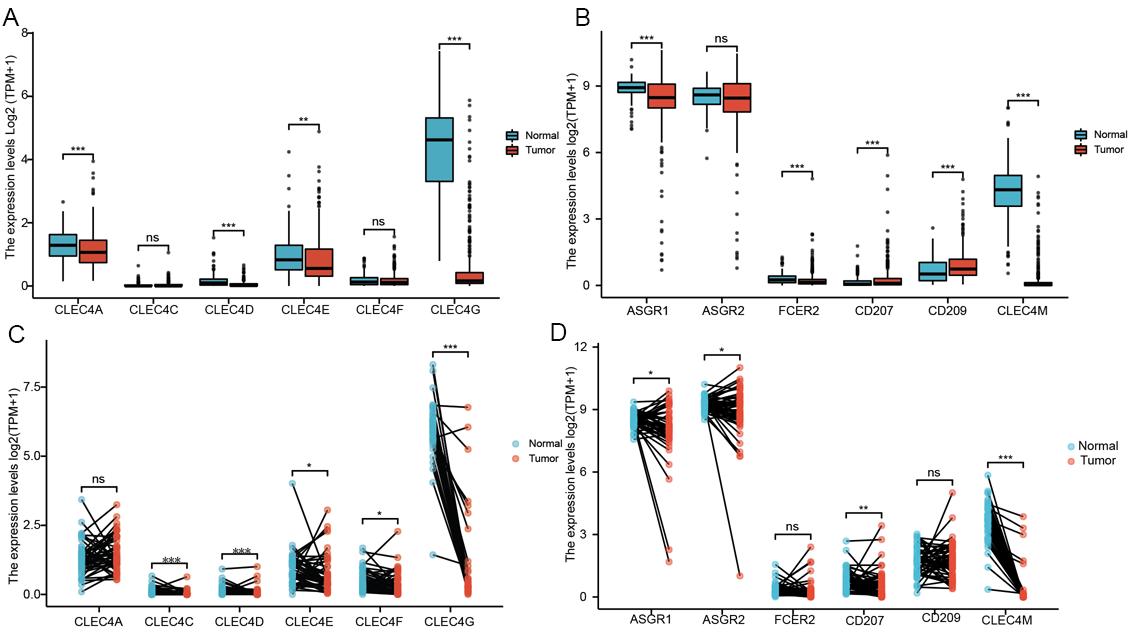

Supplement: Supplementary Figure 1 — mRNA expression level of CLEC4s in HCC compared to normal control. (A,B) Non-paired tissues, (C,D) paired tissues. ∗p< 0.05; ∗∗p< 0.01; ∗∗∗p < 0.001; ns, not significant. [file Image_1.TIF]

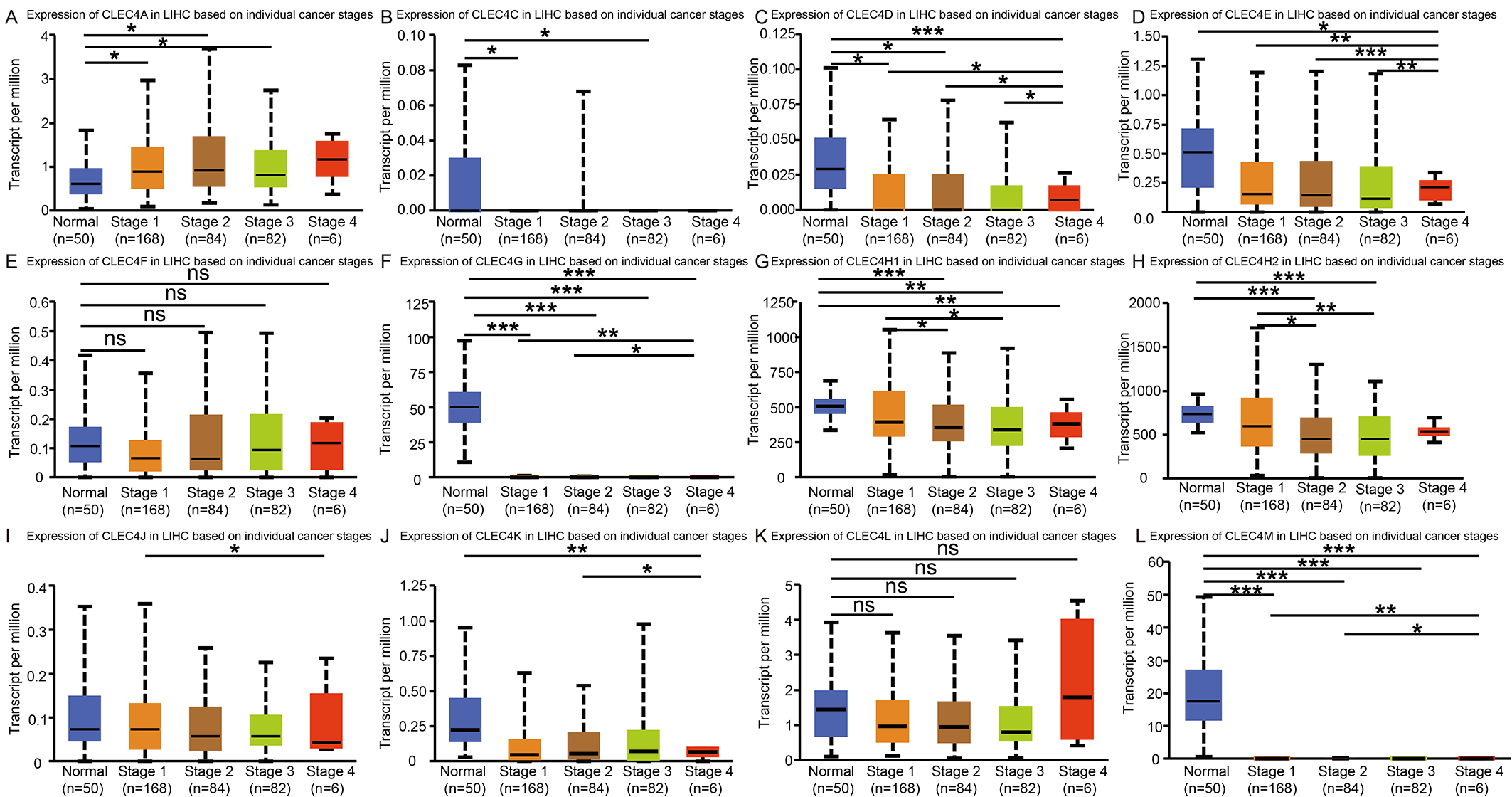

Supplement: Supplementary Figure 2 — Relationship between mRNA expression of CLEC4s and individual cancer stages of HCC patients. mRNA expressions of CLEC4s were remarkably down-regulated in HCC patients except CLEC4A. (A) CLEC4A, (B) CLEC4C, (C) CLEC4D, (D) CLEC4E, (E) CLEC4F, (F) CLEC4G, (G) CLEC4H1, (H) CLEC4H2, (I) CLEC4J, (J) CLEC4K, (K) CLEC4L, and (L) CLEC4M. ∗p < 0.05; ∗∗p< 0.01; ∗∗∗p < 0.001; ns, not significant. [file Image_2.TIF]

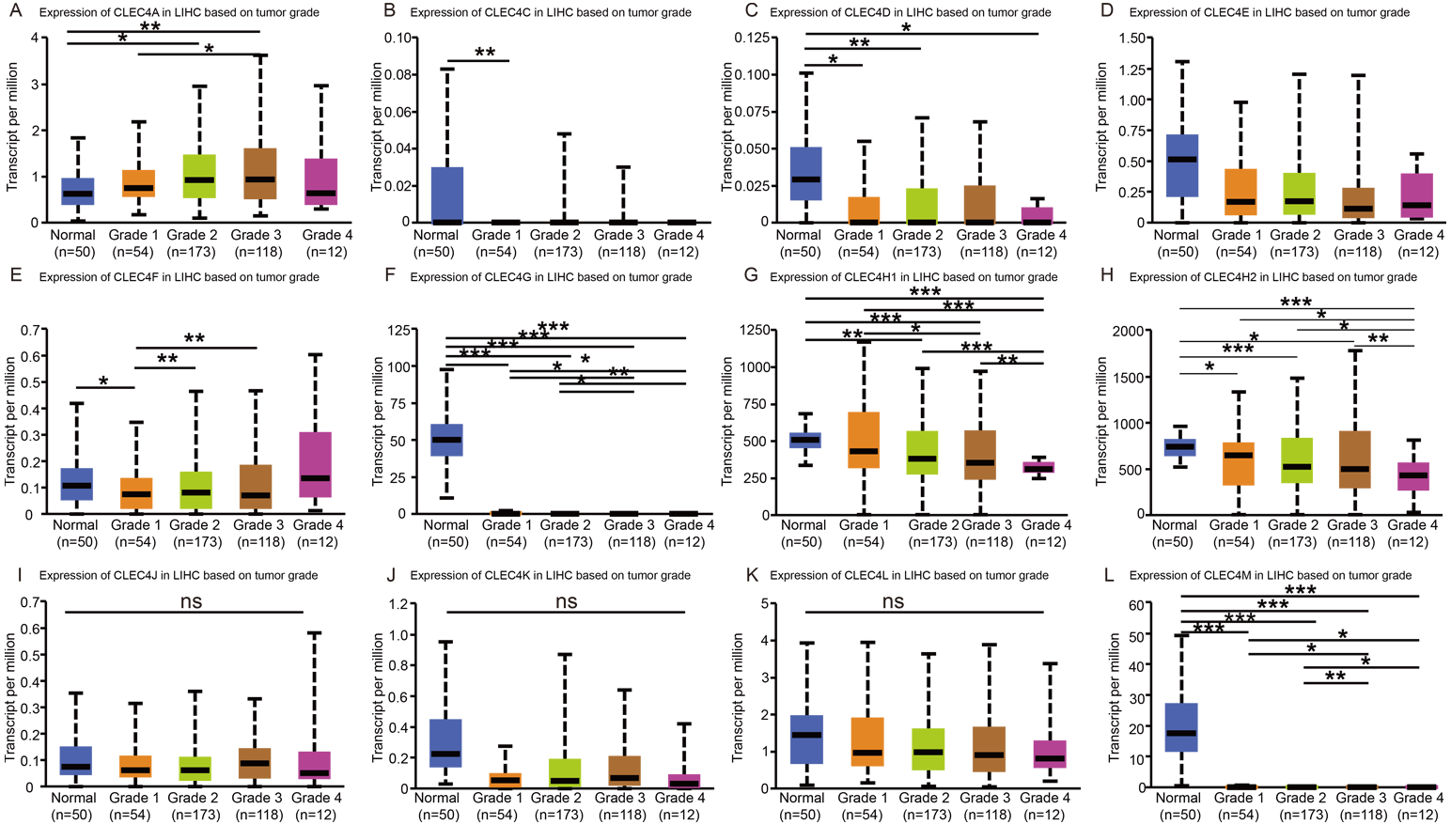

Supplement: Supplementary Figure 3 — Association of mRNA expression of CLEC4s with tumor grades of HCC patients. (A) CLEC4A, (B) CLEC4C, (C) CLEC4D, (D) CLEC4E, (E) CLEC4F, (F) CLEC4G, (G) CLEC4H1, (H) CLEC4H2, (I) CLEC4J, (J) CLEC4K, (K) CLEC4L, and (L) CLEC4M. ∗p < 0.05; ∗∗p < 0.01; ∗∗∗p < 0.001; ns, not significant. [file Image_3.TIF]

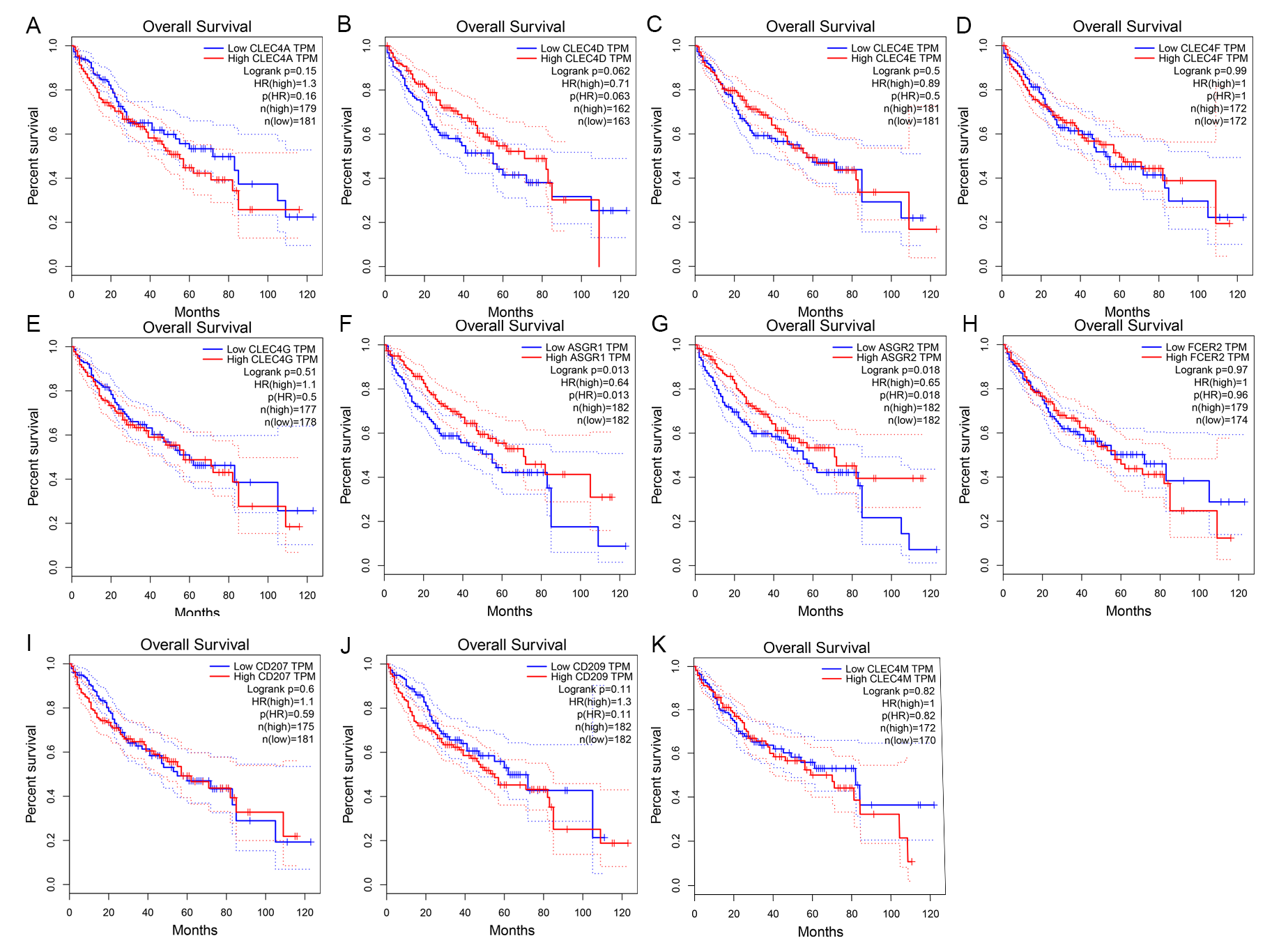

Supplement: Supplementary Figure 4 — Prognostic value of mRNA expression of CLEC4s in HCC patients (GEPIA). Higher mRNA expressions of CLEC4H1 and CLEC4H2 were significantly associated with longer overall survival of cancer patients (F,G). (A) CLEC4A, (B) CLEC4D, (C) CLEC4E, (D) CLEC4F, (E) CLEC4E, (F) CLEC4H1, (G) CLEC4H2, (H) CLEC4J, (I) CLEC4K, (J) CLEC4L, (K) CLEC4M. [file Image_4.TIF]

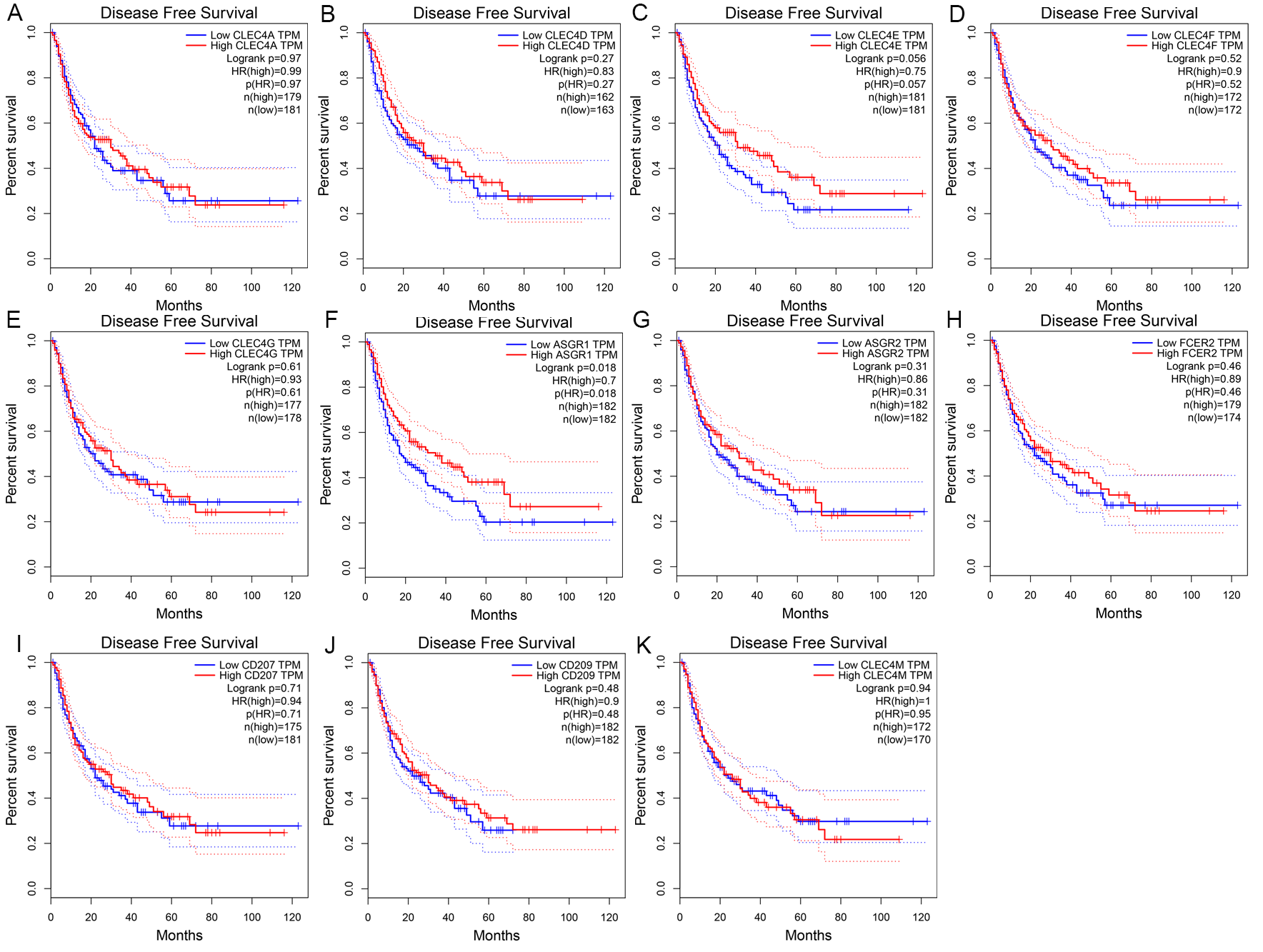

Supplement: Supplementary Figure 5 — Disease-free survival value of mRNA expression of CLEC4s in HCC patients (GEPIA). Generally, CLEC4s mRNA expression showed no correlation with disease-free survival rate in HCC. (A) CLEC4A, (B) CLEC4D, (C) CLEC4E, (D) CLEC4F, (E) CLEC4E, (F) CLEC4H1, (G) CLEC4H2, (H) CLEC4J, (I) CLEC4K, (J) CLEC4L, (K) CLEC4M. [file Image_5.TIF]

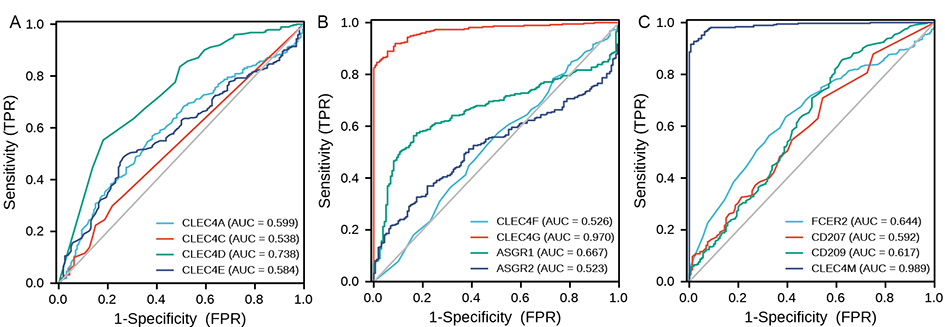

Supplement: Supplementary Figure 6 — The sensitivity and specificity of CLEC4s in HCC. The diagnostic value of CLEC4A/C/D/E (A), CLEC4F/G/H1/H2 (B), and CLEC4J/L/K/M (C) in HCC. [file Image_6.TIF]

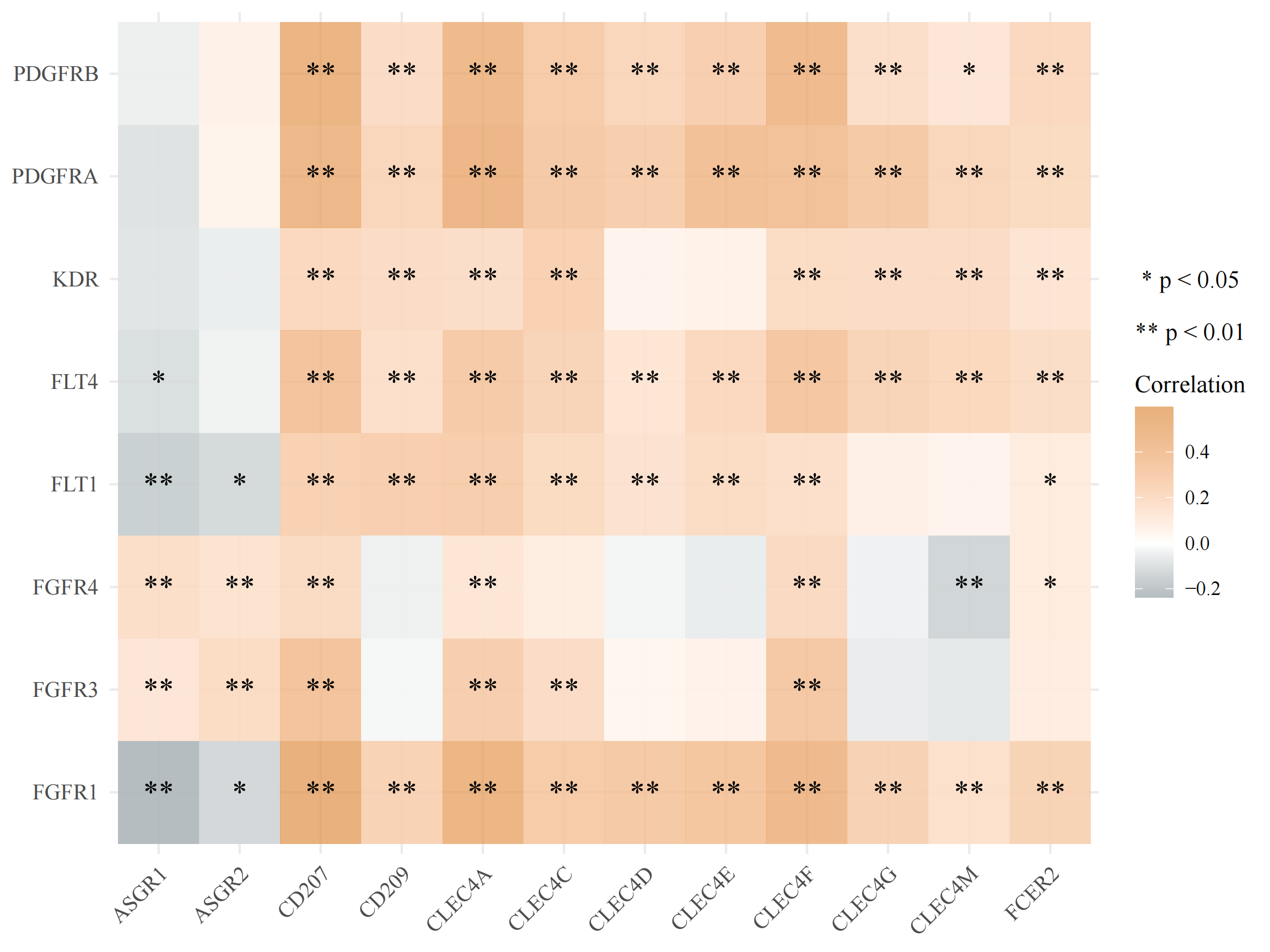

Supplement: Supplementary Figure 7 — The correlation between CLEC4s and liver cancer drug targets at mRNA level. ASGR1(CLEC4H1), ASGR2(CLEC4H2), FCER2(CLEC4J), CD207(CLEC4K), CD209(CLEC4L), VEGFR1/2/3(FLT1/KDR/FLT4), PDGFR-α/β (PDGFRA/PDGFRB). ∗p < 0.05; ∗∗p < 0.01; ns, not significant. [file Image_7.TIF]

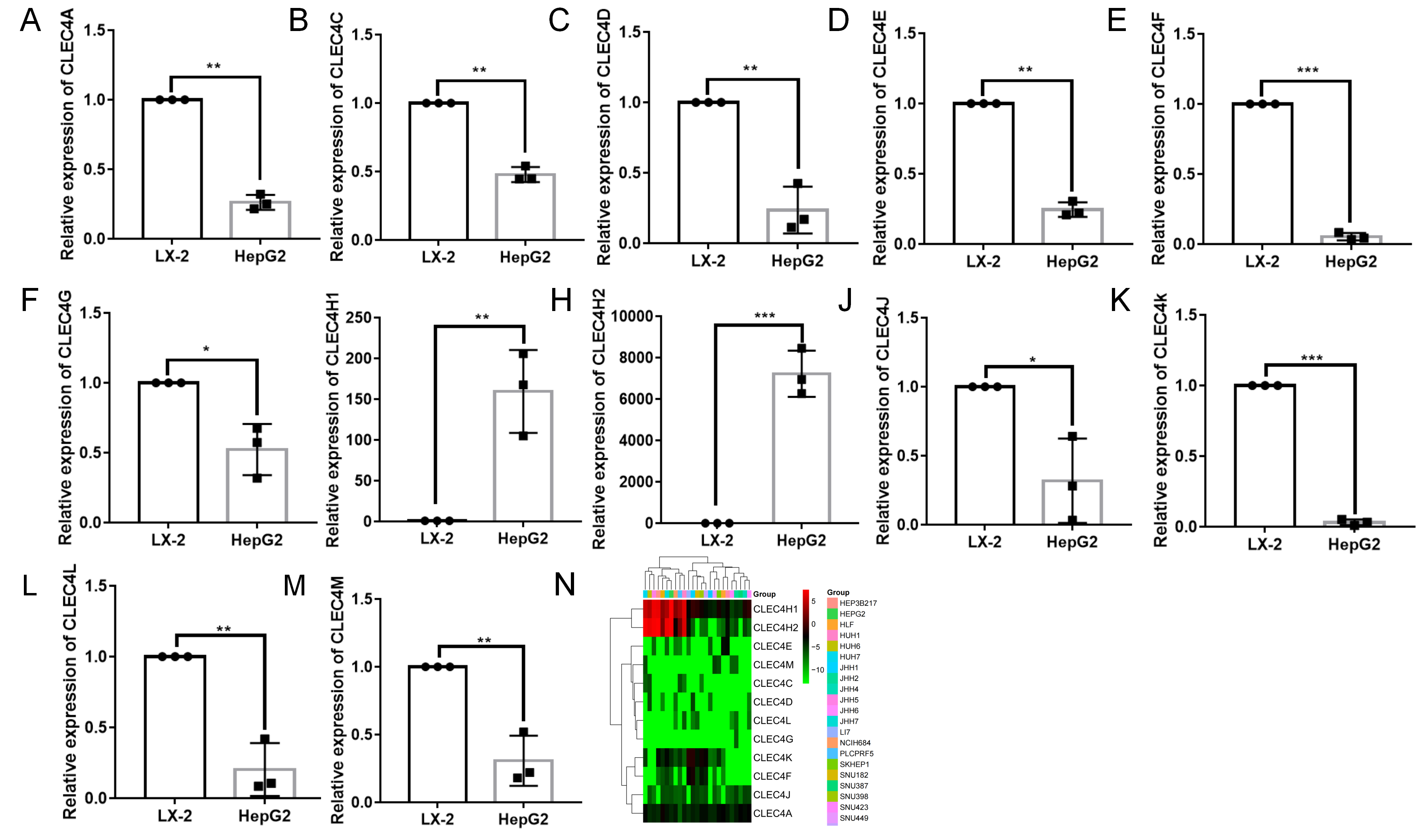

Supplement: Supplementary Figure 8 — Analysis of CLEC4 expression in liver cancer cell lines. (A–M) qPCR detected the expression level of CLEC4s in HepG2;(N)Analyze the expression level of CLEC4s in liver cancer cell lines in the CCLE database. ∗p< 0.05; ∗∗p < 0.01; ∗∗∗p < 0.001; ns, not significant. [file Image_8.TIF]

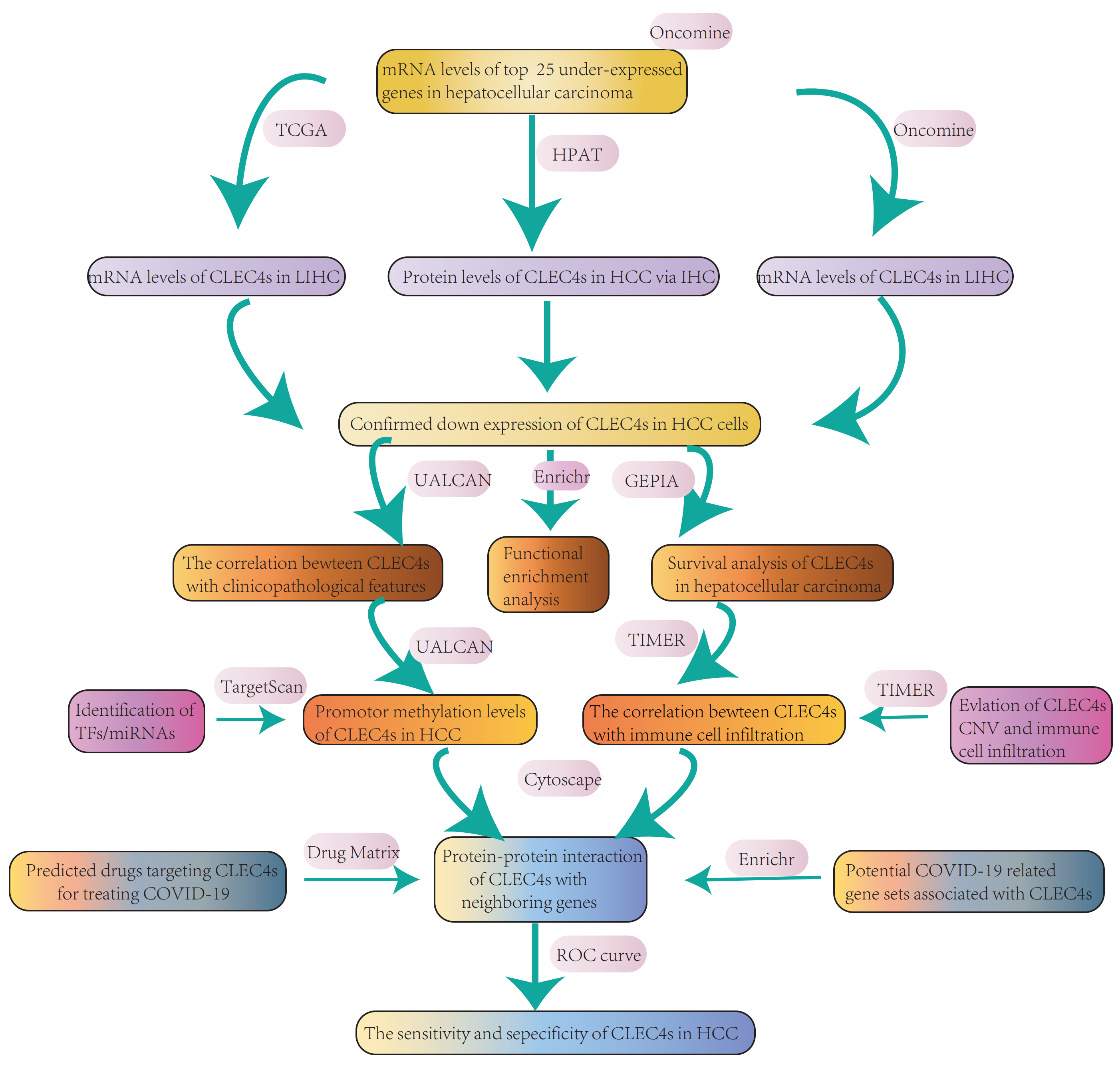

Supplement: Supplementary Figure 9 — Schematic representation of the overall workflow of this research. The flow chart summarized the overall steps for the target gene identification and analysis. [file Image_9.TIF]
