# Supplementary material for: CLEC4s as Potential Therapeutic Targets in Hepatocellular Carcinoma Microenvironment
Source: Front Cell Dev Biol. 2021 Aug 2;9:681372. doi: 10.3389/fcell.2021.681372 (PMC8367378; doi:10.3389/fcell.2021.681372)
Supplement: Supplementary Table 1 — The potential COVID-19 related gene sets associated with CLEC4. [file Table_1.DOCX]

Table S1 The potential COVID-19 related gene sets associated with CLEC4s

| Term | P-value | Odds Ratio | Combined Score |
| --- | --- | --- | --- |
| SARS 133 Literature-Associated Genes from Geneshot GeneRIF | 5.82E-05 | 18.79699 | 183.3118 |
| SARS top 50 Geneshot AutoRIF | 6.93E-05 | 37.5 | 359.1473 |
| SARS Perturbation Down Genes Mouse Lung from GSE68820:GPL7202:2 | 1.14E-04 | 31.77966 | 288.5926 |
| SARS-CoV-2/Human Interactome Gene Set from Guzzi | 4.24E-04 | 20.38043 | 158.263 |
| Up-regulated by SARS-CoV-2 in lung tissue from GSE147507 | 0.001103142 | 6.25 | 42.55995 |
| Healthy lung biopsy vs. COVID-19 infected lung series 15 from GSE147507 up genes | 0.001103142 | 6.25 | 42.55995 |
| SARS Perturbation Up Genes Mouse Lung from GSE68820:GPL7202:3 | 0.004705335 | 5.813953 | 31.15732 |
| SARS Perturbation Down Genes Mouse Lung from GSE68820:GPL7202:3 | 0.005596578 | 17.85714 | 92.6 |
| Coronavirus Perturbation Up Genes Calu-3 2B4 from GSE45042:GPL6480:2 | 0.015007903 | 5.751534 | 24.15172 |
| Down-regulated genes from COVID-19 infected bronchoalveolar lavage from patients | 0.023079187 | 4.882813 | 18.40246 |
| SARS perturbation Down Genes PBMCs GDS1028:GPL201 | 0.048140318 | 5.681818 | 17.23656 |
