# Supplementary material for: CLEC4s as Potential Therapeutic Targets in Hepatocellular Carcinoma Microenvironment
Source: Front Cell Dev Biol. 2021 Aug 2;9:681372. doi: 10.3389/fcell.2021.681372 (PMC8367378; doi:10.3389/fcell.2021.681372)
Supplement: Supplementary Table 2 — The predicted drugs targeting CLEC4s for treating COVID-19 based on Drug matrix. [file Table_2.DOCX]

Table S2 The predicted drugs targeting CLEC4s for treating COVID-19 based on Drug matrix

| Term | P-value | Odds Ratio | Combined Score |
| --- | --- | --- | --- |
| Methapyrilene-100 mg/kg in CMC-Rat-Liver-5d-dn | 8.67E-04 | 9.259259 | 65.27804 |
| Benoxaprofen-20 mg/kg in CMC-Rat-Liver-3d-dn | 9.16E-04 | 9.124088 | 63.82619 |
| Rofecoxib-775 mg/kg in Corn Oil-Rat-Liver-5d-up | 0.001019 | 8.865248 | 61.06875 |
| 4-Octylphenol-450 mg/kg in Corn Oil-Rat-Liver-3d-up | 0.001088 | 8.710801 | 59.43792 |
| Spironolactone-300 mg/kg in CMC-Rat-Liver-3d-dn | 0.001116 | 8.650519 | 58.80438 |
| 4-Nonylphenol-200 mg/kg in Corn Oil-Rat-Liver-3d-dn | 0.001131 | 8.62069 | 58.49151 |
| Oxiconazole-1500 mg/kg in Corn Oil-Rat-Liver-5d-up | 0.001145 | 8.591065 | 58.18121 |
| Ticrynafen-570 mg/kg in CMC-Rat-Liver-1d-dn | 0.001145 | 8.591065 | 58.18121 |
| Acetaminophen-486 mg/kg in Corn Oil-Rat-Liver-5d-up | 0.00116 | 8.561644 | 57.87344 |
| Procarbazine-27 mg/kg in Water-Rat-Liver-0.25d-dn | 0.00116 | 8.561644 | 57.87344 |
