# Supplementary material for: CLEC4s as Potential Therapeutic Targets in Hepatocellular Carcinoma Microenvironment
Source: Front Cell Dev Biol. 2021 Aug 2;9:681372. doi: 10.3389/fcell.2021.681372 (PMC8367378; doi:10.3389/fcell.2021.681372)
Supplement: Supplementary Table 3 — qPCR primers used in this study. [file Table_3.DOCX]

| Table S3. qPCR primers used in this study | | |
| --- | --- | --- |
| Genes | Forward primer((5' to 3')) | Reverse primer((5' to 3')) |
| 18S rRNA | TGTAACCCGTTGAACCCCATT | CCATCCAATCGGTAGTAGCG |
| CLEC4A | CTTGGCAAGACAGTGAGAAGGAC | TGACCTTCTGGATCTGAGAGCC |
| CLEC4C | GGCAGTCGTATCCATCTTGCTC | ATGACGCAGGTCAGGCTTGGAT |
| CLEC4D | GTAAGAGAGGCACAGGAGTGCA | GGAAGGCTCTCCAGTCAATAGG |
| CLEC4E | TGGACTGTCAGACCAGGTTGTC | CTCTCATGGTGGCACAGTCCTC |
| CLEC4F | CCAAGATACCGAGGCTCGTTCA | AGGCTTCGGAACAGGTCTTGTC |
| CLEC4G | ATCTGGGCAAGGTTCAGGGCTA | GCAGCATCATGACACAGTTCTCG |
| CLEC4H1 | GAAGCAGTTCGTGTCTGACCTG | AGCGAGAGAACCAGTAGCAGCT |
| CLEC4H2 | AGAACGCACACCTGGTGGTCAT | TTCCAAGAGCCATCACTGTCCG |
| CLEC4J | GGTATGCCTGTGACGACATGGA | TTCAGGTCCAAGTTCCGAAGGC |
| CLEC4K | TAATCTGCCTGACGCTGGTCCT | GGTGCTGATGTTGTCCACACGA |
| CLEC4L | GCAGTCTTCCAGAAGTAACCGC | GCTCTCCTCTGTTCCAATACTGC |
| CLEC4M | GAGTAACCGCTTCTCCTGGATG | CGCACAGTCTTCATTCCCGCTA |
